# Peripheral Artery Disease in US Primary Care Practices: A Retrospective EHR-Based Analysis from 2018 to 2022

**DOI:** 10.1007/s11606-025-10050-6

**Published:** 2025-12-08

**Authors:** Valentine C. Nriagu, Shiying Hao, Neil S. Kamdar, Eri Fukaya, Xiaoyu Wang, Karoline Kallis, David H. Rehkopf, Elsie G. Ross

**Affiliations:** 1 Department of Medicine, Maimonides Medical Center, Brooklyn, NY, USA;; 2 Center for Population Health Sciences, Stanford University School of Medicine, Stanford, CA, USA;; 3 Institute for Healthcare Policy and Innovation, University of Michigan, Ann Arbor, MI, USA;; 4 Cecil G. Sheps Center for Health Services Research, University of North Carolina, Chapel Hill, NC, USA;; 5 Department of Surgery, Division of Vascular Surgery, Stanford University School of Medicine, Stanford, CA, USA;; 6 Department of Surgery, Division of Vascular Surgery, University of California, San Diego School of Medicine, La Jolla, CA, USA;; 7 Department of Epidemiology and Population Health, Stanford University School of Medicine, Stanford, CA, USA;; 8 Department of Medicine, Division of Primary Care and Population Health, Stanford University School of Medicine, Stanford, CA, USA;; 9 Department of Pediatrics, Stanford University School of Medicine, Stanford, CA, USA;; 10 Department of Health Policy, Stanford University School of Medicine, Stanford, CA, USA;; 11 Department of Sociology, Stanford University, Stanford, CA, USA;; 12 Department of Medicine, Division of Biomedical Informatics, University of California, San Diego School of Medicine, La Jolla, CA, USA

**Keywords:** peripheral artery disease, primary care, race, socioeconomic factors, CVD

## Abstract

**BACKGROUND:**

Peripheral artery disease (PAD) affects > 10 million adults over age 40, causing high morbidity, mortality, and healthcare costs in the USA. Primary care settings are critical for early detection of PAD, and understanding the landscape of PAD diagnosis patterns can help identify high-risk populations and inform screening strategies to address disparities.

**OBJECTIVE:**

To characterize the epidemiological patterns of PAD diagnosis and examine the clinical and socioeconomic characteristics of patients diagnosed with PAD in US primary care settings.

**DESIGN:**

Retrospective cohort study utilizing electronic health record data from the American Family Cohort (AFC) from 2018 to 2022.

**SUBJECTS:**

Patients over 40 years old with at least two primary care visits during the study period.

**MAIN MEASURES:**

The primary outcome was documented PAD diagnosis. The secondary outcome was the cumulative incidence of PAD. Covariates included sociodemographic characteristics, comorbidities, body mass index, smoking status, and social deprivation index.

**RESULTS:**

A total of 18,405 patients had a new diagnosis of PAD among 2,313,650 eligible patients. PAD patients were more likely to be male (49% vs. 43%), older (64% of patients aged 60–79), current smokers (17% vs. 11%), from racial/ethnic minority groups, and residing in areas with higher social deprivation. PAD patients also had a significantly higher burden of medical comorbidities than non-PAD patients (CVD 10% vs. 3%; CAD 22% vs. 8%; heart failure 11% vs. 4%; hypertension 74% vs. 52%; diabetes 33% vs. 19%; hyperlipidemia 65% vs. 50%). Non-Hispanic Black and Hispanic individuals had higher odds of PAD diagnosis compared to non-Hispanic Whites across all comorbidity groups. Unlike CAD and hyperlipidemia with decreased incidence, PAD incidence increased notably in 2021.

**CONCLUSIONS:**

PAD diagnosis patterns across racial, ethnic, and neighborhood socioeconomic groups reveal significant differences in burden of disease. Optimized screening in the primary care setting targeting these high-risk groups is important to improve early detection and reduce PAD-related disparities.

## INTRODUCTION

Peripheral artery disease (PAD) is a common manifestation of atherosclerosis, ranking third after stroke and coronary artery disease with resultant significant morbidity and mortality.^1,2^ Over 230 million individuals are affected by PAD globally, with more than 73% being of lower socioeconomic status or living in low- and middle-income countries.^3–6^ In the United States (US), an estimated 10 to 12 million adults over 40 years old are affected and the medical care of those with PAD exceeds $21 billion annually.^5,7^ PAD prevalence increases sharply with age, from being relatively uncommon before age 50 to approximately 20% by age 80.^1,8^ The current and growing prevalence underscores the significant public health and economic burden of PAD globally and in the US.

The clinical and public health significance of PAD is evident in its strong association with major adverse cardiovascular events (MACE), including stroke, myocardial infarction, and cardiovascular death. Patients with PAD have a two- to sixfold higher risk of MACE compared to those without PAD.^5^ Additionally, PAD is strongly linked to major adverse limb events (MALE), such as critical limb ischemia including major and minor amputations with disease severity corresponding to higher rates of events.^5^ The clinical spectrum of PAD ranges from asymptomatic to limb-threatening ischemia. While 50% to 65% of individuals with PAD may be asymptomatic,^3,5,9^ their risk of MACE remains comparable to those with advanced disease.^1,2,5^ Given that many patients with PAD are asymptomatic, but can have a high cardiovascular disease burden, effective screening and appropriate early management can reduce rates of complications associated with PAD.

As an early point of contact in the health system, primary care settings play a crucial role in early detection which allows for improving PAD outcomes.^10^ Few US-based studies^11,12^ have explored the impact of primary care screening on reducing PAD burden, while in recent years, increasing research on this topic has emerged from the UK and Europe.^13,14^ The lack of primary care–centered research—exacerbated by persistent disparities, substantial knowledge gaps, and the high prevalence of asymptomatic presentations—represents a critical barrier to addressing the rising burden and inequities associated with PAD in the US.

Our study aims to characterize the epidemiological patterns of PAD diagnosis in primary care settings and examine the clinical and socioeconomic characteristics of individuals diagnosed with PAD, using nationwide longitudinal electronic health records (EHR) data. We hypothesize that social determinants of health, including area-level social deprivation, and clinical presentation may differ in ways that can inform screening strategies and highlight disparities relevant to PAD detection.

## METHODS

### Data Source

Data were obtained from the American Family Cohort (AFC), an electronic health record (EHR)-based dataset derived from the PRIME Registry. The PRIME Registry is a Qualified Clinical Data Registry (QCDR) established by the American Board of Family Medicine in 2016. It represents the largest clinical registry in the US, comprising data from over 2000 primary care practices spanning all 50 states. These practices were enrolled in the Registry based on their participation in Medicare reporting and value-based care programs, ensuring representation across diverse healthcare settings and patient populations.

The AFC dataset includes information on patient demographics, primary care visits, clinical notes, laboratory values, and other EHR-based data elements. The details have been previously documented and utilized in primary care-focused research studies.^15,16^

The study was approved by the UCSD (No. 810418) and Stanford University (No. 74468) Institutional Review Boards.

### Study Population

This retrospective cohort study included patients aged 40 years or older with at least two primary care visits between January 1, 2018, and December 31, 2022. Identification of PAD cases was based on the presence of documented PAD using validated International Classification of Diseases, 10th Revision, Clinical Modification (ICD-10-CM), diagnosis/procedure codes, or Current Procedural Terminology (CPT) codes (eTable 1). Our PAD phenotype definition was based on billing codes validated in the Phenotype KnowledgeBase (PheKB) definitions for PAD, which demonstrated a positive predictive value of 0.875–0.95 across different health systems, and was also previously validated on Stanford Health Care EHR data.^17,18^ Similarly we used validated PheKB definitions for comorbidity definitions. We required at least two distinct visits with PAD codes to increase specificity.

Patients with at least two distinct visits during which PAD was identified and documented were assigned to the PAD cohort, and all others without a PAD diagnosis or procedure code were assigned to the non-PAD cohort. PAD index date was defined as the earliest documented occurrence of PAD, identified using either ICD or CPT codes. Non-PAD index date was designated as the last recorded visit within the study period to maximize the lookback period for assessing clinical comorbidity history. To ensure the identification of potential incident PAD cases within the study period, patients with a documented PAD diagnosis prior to January 1, 2018, or after December 31, 2022, were excluded from the PAD cohort. Additionally, patients younger than 40 at the index date or those with fewer than two visits within the timeframe of 1 year to 60 days before the index date were excluded from the analysis.

### Covariates and Outcomes

The primary outcome for this study was the documentation of PAD through diagnosis or procedure codes (eTable 1) at any time during the study period. The secondary outcome was the date of the first recorded PAD diagnosis, which was used to determine the annual incidence.

We categorized our predictors and covariates into the following groups: demographic characteristics (sex, age, race, and ethnicity), medical comorbidities (cerebrovascular disease (CVD), coronary artery disease (CAD), heart failure (HF), hypertension (HTN), diabetes mellitus (DM), hyperlipidemia (HLD)), and additional risk factors such as body mass index (BMI), and smoking status, using PheKB and previously established definitions.^18^ Additionally, we incorporated geographic and socioeconomic factors, including the U.S. Census Tract geographic location, and the Social Deprivation Index (SDI), which was calculated at the census tract level for all census tracts in the US.^19^ Rural–urban commuting area (RUCA) categories were determined based on five-digit zip code data.^20^

In our analysis, missing data were handled differently for categorical and numerical data elements. Patients with missing age were excluded from the study population. For categorical characteristics including race/ethnicity, gender, smoking status, census region, and rural–urban-commuting area, an “Unknown” category was used to capture missing values. Missing BMI values were set to NULL, and patients without BMI data were excluded from the analysis exploring the relationship between BMI and PAD.

### Statistical Analysis

We conducted a retrospective, descriptive analysis to evaluate the associations between patient-level characteristics and PAD diagnosis. The distribution of predictor variables and covariates was summarized for the overall study population, as well as separately for the PAD cohort and non-PAD cohort.

To compare patient-level characteristics between the PAD and non-PAD cohorts, we employed Chi-squared tests for categorical variables and nonparametric Wilcoxon tests for continuous variables. Additionally, within each race and ethnicity category, age-adjusted odds ratios (ORs) were calculated for each patient-level characteristic to assess associations with PAD. For patients identified as non-Hispanic whites, non-Hispanic Blacks, or Hispanic, an age-adjusted, multivariable analysis was conducted to examine the associations between comorbidities, race/ethnicity, and their relationship with PAD diagnosis to evaluate whether certain comorbidities were more (or less) likely to be associated with different racial/ethnic groups. We used three racial/ethnic groups with the largest patient numbers to ensure adequate statistical power.

Our multivariable logistic regression models included clinically relevant covariates: age, race, and ethnicity, one of six chronic conditions (CVD, CAD, HF, HTN, diabetes, and HLD), and the interaction between the chronic condition and race/ethnicity. For example, one multivariable model incorporated CVD, age, and race/ethnicity as main effects, along with an interaction term between CVD and race/ethnicity. In this case, the interaction term was included to assess both between-group and within-group effects of race/ethnicity and CVD on PAD diagnosis, allowing for the estimation of adjusted odds ratios. In this model, race and CVD, as joint exposures, were age-adjusted to estimate the odds and 95% confidence intervals for documented PAD diagnosis. Similar models were constructed for each of the six chronic conditions (CVD, CAD, HF, HTN, diabetes, and HLD).

The annual cumulative incidence of PAD, our secondary outcome, was calculated using the following approach. The incidence denominator comprised patients who had at least one primary care visit during the calendar year, were aged 40 years or older by the end of that year and had no documented PAD diagnosis at the beginning of the same year. The numerator included patients who received a PAD diagnosis documented in at least two separate visits, with the first PAD diagnosis documented within the year and were aged 40 years or older at the time of their first documented PAD diagnosis. The incidence was standardized through direct age adjustment using the reference age distribution from the 2022 U.S. Census. A similar methodology was applied to determine the annual cumulative incidence of CAD and HLD. All analyses were conducted using Python (Version 3.7.10).

## RESULTS

A total of 2,313,650 patients met study eligibility criteria during the study period, of whom 18,405 (0.8%) had a documented diagnosis of PAD, while 2,295,245 (99.2%) did not (eFigure 1).

Among patients in the PAD cohort, approximately 64% were between the ages of 60 and 79, with the highest proportion aged 70–79 years (36%), followed by those aged 60–69 years (28%). The PAD cohort had a higher proportion of males compared to the non-PAD cohort (49% vs. 43%). The PAD cohort also had a greater representation of non-Hispanic Black patients (10% vs. 7%) and Hispanic patients (10% vs. 7%) compared to the non-PAD cohort. Additionally, the proportion of smokers was significantly higher in the PAD cohort (17% vs. 11%). When comparing the prevalence of comorbidities, the PAD cohort consistently exhibited a higher disease burden than the non-PAD cohort (CVD 10% vs. 3%; CAD 22% vs. 8%; HF 11% vs. 4%; HTN 74% vs. 52%; diabetes 33% vs. 19%; HLD 65% vs. 50%). A broader description of both cohorts is presented in Table 1.

We conducted a sub-group analysis stratified by age, gender, BMI, and smoking status to examine differences by race and ethnicity among patients with and without a PAD diagnosis (Table 2, eTable 2). Across all racial and ethnic groups, PAD patients were significantly older than their non-PAD counterparts. After adjusting for age, female gender was generally associated with a lower likelihood of PAD diagnosis compared to males across all racial and ethnic groups, except among American Indian or Alaska Native individuals, where no significant difference was observed. Conversely, current smoking was associated with higher odds of PAD diagnosis across all racial and ethnic groups, except for American Indian or Alaska Native and Native Hawaiian or Other Pacific Islander populations. BMI was associated with an increased likelihood of PAD diagnosis only among non-Hispanic White and Hispanic individuals.

We conducted analyses to assess the possible effect modification of race and ethnicity on the association between chronic conditions and the odds of PAD diagnosis. Comorbidities were strongly associated with PAD diagnosis across all groups. Additionally, the presence of one or more chronic conditions was significantly associated with increased odds of PAD diagnosis across all three racial and ethnic categories (Table 3). However, non-Hispanic Blacks and Hispanic individuals had higher odds of PAD diagnosis compared to their non-Hispanic White counterparts, regardless of the presence of chronic conditions (Table 4). The adjusted odds ratios for both between-group and within-group comparisons of race/ethnicity and chronic conditions are presented in eTable 3, Table 3, and Table 4. Sensitivity analyses using log-binomial regression to directly estimate risk ratios yielded consistent results with those from logistic regression (eTable 4 and eTable 5).

Significant differences in social determinants of health were observed between PAD and non-PAD patients. PAD patients had higher census tract level SDI scores (mean [SD], 51 [28, 73] vs. 42 [20, 65]), indicating lower area-level socioeconomic status, compared to their non-PAD counterparts (Table 1). Additionally, variations in social determinants of health were noted both between and within racial and ethnic groups among PAD and non-PAD patients (eTable 6, eTable 7, eFigure 2).

Lastly, we estimated the age-adjusted annual cumulative incidence of PAD, CAD, and HLD per 1000 patients (eFigure 3). The annual incidence of PAD in the AFC remained stable from 2018 to 2020, followed by a notable increase in 2021 (counts per 1000 and 95% CI in 2021 vs 2020: 2.8 [2.7, 2.9] vs. 2.2 [2.1, 2.3]). As a comparison, we evaluated the incidence of other associated chronic diseases. We found that the annual incidence of HLD steadily declined from 70.7 (95% CI 70.2, 21.1) in 2018 to 48.3 (95% CI 47.8, 48.8) in 2022. In contrast, the annual incidence of CAD fluctuated modestly, showing a 13.7% decline over the study period, from 9.5 (95% CI 9.4, 9.7) per 1000 in 2018 to 8.2 (95% CI 8.0, 8.3) per 1000 in 2022.

## DISCUSSION

Our study provides insights into the clinical and socioeconomic patterns of PAD diagnosis among individuals aged 40 years and older observed in primary care settings across the US. The findings underscore the substantial burden of PAD, its strong association with comorbidities, and the significant disparities in PAD incidence across racial, ethnic, and neighborhood socioeconomic groups.

A critical finding of our study is the substantial disease burden of co-morbid conditions among PAD patients, who demonstrated significantly higher rates of cardiovascular comorbidities, including CVD, CAD, HF, HTN, diabetes, and hyperlipidemia, compared to their non-PAD counterparts. These findings align with previous research, which has established PAD as a major manifestation of systemic atherosclerosis, often coexisting with other cardiovascular diseases and contributing to increased morbidity and mortality.^6^ The elevated prevalence of cardiovascular comorbidities among PAD patients underscores the need for comprehensive cardiovascular risk assessment and management strategies within primary care settings. In particular, patients with a diagnosis of other cardiovascular and cardiometabolic diseases should be considered for screening.

We also observed notable demographic differences in PAD diagnosis, with non-Hispanic Black and Hispanic individuals exhibiting higher odds of PAD diagnosis compared to non-Hispanic White individuals, even after adjusting for comorbidities. This finding is consistent with previous studies that have identified Black race as an independent risk factor for PAD.^20^ The higher PAD incidence among Black and Hispanic populations may be driven by a complex interplay of genetic predisposition, increased prevalence of traditional cardiovascular risk factors, and disparities in healthcare access, preventive care, and treatment.^21,22^ Addressing these disparities requires targeted interventions, including improved access to early screening, culturally tailored education, and community-based prevention programs to mitigate the disproportionate burden of PAD in these populations.

Our analysis reveals differences in areal-level socioeconomic patterns between PAD and non-PAD patients. Our study further highlights the significant role of social determinants of health in PAD incidence. PAD patients had significantly higher SDI scores compared to non-PAD patients, indicating greater residence in more socioeconomically disadvantaged neighborhoods. Previous studies have demonstrated that personal lower socioeconomic status can be associated with higher rates of cardiovascular disease, limited access to preventive care, and worse health outcomes.^23^ Here, we demonstrate that neighborhood dynamics may also contribute specifically to PAD risk. Thus, addressing disparities in PAD rates may require healthcare policies that prioritize social determinants of health, including expanded healthcare access, community-based interventions, and improved public health initiatives targeting high-risk populations.

The annual cumulative incidence of PAD in our study remained stable from 2018 to 2020 but showed a notable increase in 2021. This surge may be attributable to disruptions in routine healthcare services during the COVID-19 pandemic, leading to delayed diagnoses and subsequent identification of PAD cases as healthcare utilization rebounded. However, we did not see the same increase in other chronic conditions in 2021 such as HLD or CAD in this primary care setting. Thus, the increase may align with emerging evidence that demonstrates that SARS-CoV-2 infection accelerates atherosclerotic progression beyond acute thrombotic events. A 2023 Nature Cardiovascular Research study found that SARS-CoV-2 directly infects atherosclerotic plaques, triggering pro-atherogenic inflammatory cascades.^24^ The CARTESIAN multicenter study documented persistent vascular stiffening over 6 months post-infection.^25^ Additionally, large cohort studies from the UK have demonstrated the increased arterial, as well as venous, thrombosis risk from COVID infection.^26^ The combination of mechanisms—including ACE2 downregulation, sustained inflammation, oxidative stress, and increased thrombosis—represents both chronic atherosclerotic acceleration and acute thrombosis, which may have increased symptoms in patients with previously asymptomatic disease, and may explain the increase in PAD diagnoses in the primary care setting post-2020 that we observed.

Our study leverages a large, diverse dataset from the American Family Cohort (AFC), providing robust real-world insights into PAD epidemiology in primary care settings across the US. The inclusion of a broad patient population enhances the generalizability of our findings. Additionally, the use of EHR data allows for a longitudinal assessment of PAD incidence and trends, providing valuable insights into disease progression and healthcare utilization patterns and opportunities for enhanced screening in vulnerable populations.

Even so, our study has some limitations. First, while our PAD phenotype definition is based on validated algorithms, we did not perform local validation within the AFC dataset as we did not have ready access to more objective data such as ABIs for validation assessment. We acknowledge that documentation and coding practices can vary across providers and practices, potentially introducing misclassification bias. Second, our findings may be subject to informed presence bias, where patients with greater healthcare engagement are more likely to receive a PAD diagnosis. We attempted to mitigate this by requiring at least two visits for all patients. Third, we are unable to capture all confounders that may influence PAD risk, such as disease severity, medication adherence, health literacy, and individual-level socioeconomic factors. Fourth, area-level SDI measures are subject to administrative boundaries that may not correspond to meaningful social neighborhoods as experienced by residents.

Additionally, we only capture primary care utilization for the patients in practices that contribute cases within the specific calendar year. We do not capture information about the patient if they visit other practices that are not within the registry, nor any specialist care or referrals. Thus, PAD incidence may be underestimated if diagnosed elsewhere. Lastly, our observational study design precludes causal inferences regarding the relationships between PAD, comorbidities, and social determinants of health. Future studies employing time-to-event analysis with hazard models would provide additional insights into the temporal dynamics of PAD development.

Despite these disadvantages, our dataset includes a comprehensive representation of the US population, spanning multiple geographies. Utilizing EHR data from the largest primary healthcare registry enabled us to identify the contemporary persistence of demographic and socioeconomic differences in PAD disease burden and the overall increase in disease incidence after the COVID-19 pandemic.

## CONCLUSION

Our findings highlight the significant burden of PAD, its strong association with cardiovascular and metabolic comorbidities, and the pronounced disparities in PAD diagnosis patterns across racial, ethnic, and neighborhood socioeconomic groups. We also identified that PAD, unlike other comorbid conditions such as HLD and CAD, has had an increase in incidence following the COVID-19 pandemic, potentially related to the vascular atherosclerotic and thrombotic effects of the SARS-CoV-2 infection. Given that primary care settings play a critical role in early detection, risk factor modification, and equitable healthcare delivery, future research should focus on optimizing screening strategies, addressing healthcare disparities, and implementing targeted interventions to help decrease the incidence of PAD and improve the overall medical and surgical management of PAD patients. In particular, given the higher burden of disease in Black and Hispanic populations, and residents of socioeconomically disadvantaged neighborhoods, strategies to improve care will need to be culturally tailored and address structural barriers to healthcare access.

## Supplementary Material

Supplementary material

**Supplementary Information** The online version contains supplementary material available at https://doi.org/10.1007/s11606-025-10050-6.

## Figures and Tables

**Table 1:** Sociodemographic Characteristics of Primary Care Patients with Peripheral Artery Disease (*PAD*) and Non-PAD Diagnosis Codes: American Family Cohort, Jan 1, 2018, to Dec 31, 2022

Characteristics	All	PAD	Non-PAD	*P* value
Patient counts, *n*	2,313,650	18,405	2,295,245	
Age group, *n (%)*				< 0.001***
40–49	392,062 (17)	480 (3)	391,582 (17)	
50–59	525,923 (23)	1815 (10)	524,108 (23)	
60–69	612,540 (27)	5173 (28)	607,367 (27)	
70–79	485,425 (21)	6639 (36)	478,786 (21)	
≥ 80	297,700 (13)	4298 (23)	293,402 (13)	
Race/ethnicity, *n* (%)				< 0.001***
Non-Hispanic Black/African American	158,746 (7)	1918 (10)	156,828 (7)	
Non-Hispanic White	1,584,560 (69)	12,374 (67)	1,572,186 (69)	
Hispanic	170,758 (7)	1746 (10)	169,012 (7)	
Asian	41,886 (2)	253 (1)	41,633 (2)	
American Indian or Alaska Native	10,578 (0.5)	74 (0.4)	10,504 (0.5)	
Native Hawaiian or other Pacific Islander	4,367 (0.2)	37 (0.2)	4330 (0.2)	
Other/unknown	342,755 (15)	2003 (11)	340,752 (15)	
Gender, *n* (%)				< 0.001***
Female	1,315,300 (57)	9325 (51)	1,305,975 (57)	
Male	997,212 (43)	9077 (49)	988,135 (43)	
Other/unknown	1138 (0)	3 (0)	1135 (0)	
Chronic conditions, *n* (%)				
Cerebrovascular disease (CVD)	78,937 (3)	1785 (10)	77,152 (3)	< 0.001***
Coronary artery disease (CAD)	183,136 (8)	4070 (22)	179,066 (8)	< 0.001***
Heart failure (HF)	96,053 (4)	1957 (11)	94,096 (4)	< 0.001***
Hypertension (HTN)	1,207,829 (52)	13,557 (74)	1,194,272 (52)	< 0.001***
Diabetes	453,552 (20)	6150 (33)	447,402 (19)	< 0.001***
Hyperlipidemia (HLD)	1,163,063 (50)	12,051 (65)	1,151,012 (50)	< 0.001***
Body Mass Index (BMI), mean (SD)	30.3 (6.6)	29.9 (6.3)	30.3 (6.6)	0.06
Smoking status, *n* (%)				< 0.001***
Current smokers	246,658 (11)	3203 (17)	243,455 (11)	
Non-smokers	1,774,597 (77)	12,105 (66)	1,762,492 (77)	
Other/unknown	292,395 (13)	3097 (17)	289,298 (13)	
US Census Region, *n* (%)				< 0.001***
Midwest	400,415 (17)	2148 (12)	398,267 (17)	
Northeast	254,938 (11)	1153 (6)	253,785 (11)	
South	1,252,912 (54)	12,369 (67)	1,240,543 (54)	
West	389,259 (17)	2670 (15)	386,589 (17)	
Unknown	16,126 (1)	65 (0)	16,061 (1)	
Social Deprivation, median (IQR)				
Social Deprivation Index (SDI) at census tract level	42 (20, 65)	51 (28, 73)	42 (20, 65)	< 0.001***
Rural–Urban-Commuting Area (RUCA) at zip code level, *n* (%)				< 0.001***
Metropolitan areas	1,652,550 (71.4)	13,285 (72)	1,639,265 (71)	
Micropolitan areas	342,696 (14.8)	2574 (14)	340,122 (15)	
Small town	187,427 (8.1)	1356 (7)	186,071 (8)	
Rural areas	114,781 (5)	1123 (6)	113,658 (5)	
Unknown	16,196 (0.7)	67 (0)	16,129 (1)	

**Table 2:** Age-Adjusted Odds Ratios and 95% Confidence Intervals (CI) for Peripheral Artery Disease (PAD) Compared to Non-PAD by Gender, Body Mass Index (BMI), and Smoking Status: American Family Cohort, Jan 1, 2018, to Dec 31, 2022 (*n* = 2,313,650)

Race and ethnicity	Odds Ratio (95% CI)^#^			
	Age	Female	Body Mass Index (BMI)^##^	Current Smokers
Non-Hispanic Black or African American	1.05 (1.05,1.05)***	0.81 (0.74,0.89)***	0.99 (0.96,1.00)	2.20 (1.94,2.49)***
Non-Hispanic White	1.05 (1.04,1.05)***	0.72 (0.69,0.74)***	1.01 (1.00,1.02)*	2.95 (2.81,3.09)***
Hispanic, Odds Ratio (95% CI)	1.06 (1.06,1.07)***	0.82 (0.75,0.90)***	1.06 (1.02,1.10)**	1.96 (1.65,2.34)***
Asian, Odds Ratio (95% CI)	1.05 (1.04,1.06)***	0.75 (0.59,0.97)*	1.00 (0.93,1.07)	3.06 (2.00,4.70)***
American Indian or Alaska Native, Odds Ratio (95% CI)	1.05 (1.03,1.07) ***	0.70 (0.44,1.10)	NA	1.08 (0.58,2.01)
Native Hawaiian or Other Pacific Islander, Odds Ratio (95% CI)	1.05 (1.02,1.07)***	0.41 (0.21,0.81)**	1.01 (0.91,1.11)	2.41 (0.96,6.08)

* *P* < 0.05,

** *P* < 0.01,

*** *P* < 0.001

# All odds ratios for all characteristics, except age, are adjusted for age. For example, the odds ratio for female was calculated by a logistic regression with the following model: Outcome (PAD vs. no PAD) ~ age + female (yes or no)

## For PAD patients, BMI data were missing for 95%, 93%, 96%, 85%, 100%, and 78% of individuals in the non-Hispanic Black or African American, non-Hispanic White, Hispanic, Asian, American Indian or Alaska Native, and Native Hawaiian or other Pacific Islander groups, respectively. Among non-PAD patients, BMI data were missing for 92%, 92%, 94%, 91%, 96%, and 85% of individuals in the corresponding groups

**Table 3:** Age-Adjusted Odds Ratios and 95% Confidence Intervals (*CI*) for Peripheral Artery Disease (*PAD*) by Chronic Conditions Across Race and Ethnicity Categories: American Family Cohort, Jan 1, 2018, to Dec 31, 2022 (*n* = 2,313,650)

Chronic conditions^#^	Non-Hispanic Black or African American		Non-Hispanic White		Hispanic	
	Odds ratio (95% CI)^##^	*P* value	Odds ratio (95% CI)	*P* value	Odds ratio (95% CI)	*P* value
CVD vs. non-CVD	1.70 (1.45, 2.01)	< 0.001***	2.02 (1.90, 2.14)	< 0.001***	1.77 (1.46, 2.16)	< 0.001***
CAD vs. non-CAD	2.18 (1.93, 2.46)	< 0.001***	2.37 (2.27, 2.48)	< 0.001***	2.11 (1.86, 2.40)	< 0.001***
HF vs. non-HF	1.42 (1.23, 1.64)	< 0.001***	1.64 (1.55, 1.74)	< 0.001***	1.79 (1.53, 2.10)	< 0.001***
HTN vs. non-HTN	2.39 (2.10, 2.73)	< 0.001***	1.72 (1.66, 1.80)	< 0.001***	2.11 (1.89, 2.36)	< 0.001***
Diabetes vs. non-diabetes	1.63 (1.49, 1.79)	< 0.001***	1.78 (1.71, 1.85)	< 0.001***	1.68 (1.53, 1.85)	< 0.001***
HLD vs. non-HLD	1.49 (1.36, 1.64)	< 0.001***	1.47 (1.41, 1.52)	< 0.001***	1.36 (1.23, 1.50)	< 0.001***

*P* values were adjusted using the Benjamini–Hochberg procedure.

* *P* < 0.05,

** *P* < 0.01,

*** *P* < 0.001

# Chronic conditions include cerebrovascular disease (*CVD*), coronary artery disease (*CAD*), heart failure (*HF*), hypertension (*HTN*), diabetes, and hyperlipidemia (*HLD*)

## Odds ratios were calculated from a multivariate logistic regression with predictors of age, race and ethnicity category, a chronic condition, and an interaction between the race and ethnicity category and the chronic condition. For example, the odds ratio for CVD vs. non-CVD was obtained using the following model: outcome (PAD vs. non-PAD) ~ age + race/ethnicity + CVD + race/ethnicity × CVD. The odds ratio of 1.70 for CVD means that within the non-Hispanic Black or African American category, the odds of having PAD for an individual with CVD were 1.7 times greater than the odds of having PAD for an individual without CVD

**Table 4:** Age-Adjusted Odds Ratios and 95% Confidence Intervals (*CI*s) for Peripheral Artery Disease (*PAD*) by Each of the Chronic Conditions Across Race and Ethnicity Categories: American Family Cohort, Jan 1, 2018, to Dec 31, 2022 (*n* = 2,313,650)

Chronic conditions^#^	Non-Hispanic Black or African American vs. non-Hispanic White		Hispanic vs. non-Hispanic White		Hispanic vs. non-Hispanic Black or African American	
	Odds ratio (95% CI)^##^	*P* value	Odds ratio (95% CI)	*P* value	Odds ratio (95% CI)	*P* value
Non-CVD	1.83 (1.72, 1.95)	< 0.001***	1.65 (1.55, 1.76)	< 0.001***	0.90 (0.83, 0.98)	0.03*
CVD	1.55 (1.27, 1.89)	< 0.001***	1.45 (1.15, 1.84)	0.004 **	0.94 (0.70, 1.26)	0.9
Non-CAD	1.89 (1.77, 2.01)	< 0.001***	1.69 (1.58, 1.80)	< 0.001***	0.89 (0.82, 0.97)	0.02*
CAD	1.74 (1.51, 2.00)	< 0.001***	1.50 (1.30, 1.74)	< 0.001***	0.87 (0.71, 1.05)	0.5
Non-HF	1.82 (1.71, 1.93)	< 0.001***	1.60 (1.51, 1.71)	< 0.001***	0.88 (0.81, 0.96)	0.006**
HF	1.57 (1.32, 1.87)	< 0.001***	1.75 (1.44, 2.12)	< 0.001***	1.11 (0.87, 1.42)	0.9
Non-HTN	1.27 (1.09, 1.48)	0.006 **	1.36 (1.21, 1.54)	< 0.001***	1.08 (0.89, 1.30)	0.9
HTN	1.76 (1.65, 1.87)	< 0.001***	1.67 (1.56, 1.79)	< 0.001***	0.95 (0.87, 1.04)	0.7
Non-diabetes	1.73 (1.61, 1.87)	< 0.001***	1.52 (1.40, 1.65)	< 0.001***	0.88 (0.79, 0.97)	0.04*
Diabetes	1.59 (1.45, 1.74)	< 0.001***	1.44 (1.31, 1.58)	< 0.001***	0.91 (0.81, 1.02)	0.4
Non-HLD	1.80 (1.63, 1.98)	< 0.001***	1.68 (1.51, 1.87)	< 0.001***	0.94 (0.82, 1.07)	0.9
HLD	1.83 (1.70, 1.97)	< 0.001***	1.56 (1.45, 1.68)	< 0.001***	0.85 (0.77, 0.94)	0.001**

*P* values were adjusted using the Benjamini–Hochberg procedure.

* *P* < 0.05,

** *P* < 0.01,

*** *P* < 0.001

# Chronic conditions include cerebrovascular disease (*CVD*), coronary artery disease (*CAD*), heart failure (*HF*), hypertension (*HTN*), diabetes, and hyperlipidemia (*HLD*)

## Odds ratios were calculated from a multivariate logistic regression with predictors of age, race and ethnicity category, a chronic condition, and an interaction between the race and ethnicity category and the chronic condition. For example, the odds ratio for Non-Hispanic Black or African American vs. Non-Hispanic White under a condition “Non-CVD” was obtained using the following model: outcome (PAD vs. non-PAD) ~ age + race/ethnicity + CVD + race/ethnicity × CVD. The odds ratio of 1.83 means that among individuals without a history of CVD, the odds of having PAD for a non-Hispanic Black or African American individual were 1.83 times greater than the odds of having PAD for a non-Hispanic White individual
