# Supplementary material for: Peripheral Artery Disease in US Primary Care Practices: A Retrospective EHR-Based Analysis from 2018 to 2022
Source: J Gen Intern Med. Author manuscript; Available in PMC 2026 Jul 22. (PMC13390906; doi:10.1007/s11606-025-10050-6)

**eTable 1. ICD-9 and ICD-10 Diagnosis Codes (Inclusion and Exclusion) and Procedure Codes (Inclusion Only) Used to Define Peripheral Artery Disease (PAD)**

| **Diagnosis/Procedure Codes for Inclusion** | **Diagnosis Codes for Exclusion** |
| --- | --- |
| **Diagnosis**  ICD-9-CM: 440.2, 440.21, 440.22, 440.23, 440.24, 440.29, 440.3, 440.31, 440.32, 440.4, 440.8, 443.9 ICD-10-CM: I70.20, I70.21, I70.22, I70.23, I70.24, I70.25, I70.26, I70.29, I70.30, I70.31, I70.32, I70.33, I70.34, I70.35, I70.36, I70.39, I70.40, I70.41, I70.42, I70.43, I70.44, I70.45, I70.46, I70.49, I70.50, I70.51, I70.52, I70.53, I70.54, I70.55, I70.56, I70.59, I70.60, I70.61, I70.62, I70.63, I70.64, I70.65, I70.66, I70.69, I70.70, I70.71, I70.72, I70.73, I70.74, I70.75, I70.76, I70.79, I70.8, I70.92, I73.9  **Procedure** ICD-9-CM: 38.18, 39.5, 39.9, 84.1, 84.11, 84.12, 84.13, 84.14, 84.15, 84.16, 84.17, 84.18, 84.19 ICD-10-PCS: 04100J7, 04100J8, 04100JF, 04100JG, 04100JH, 04100JJ, 04100JK, 041C0JD, 041C0JH, 041D0JF, 041D0JJ, 041H0JH, 041H0KJ, 041H4JH, 041J0JJ, 041J4JJ, 041K0JH, 041K0JJ, 041K0JK, 041K0JL, 041K0JM, 041K0JN, 041K0JP, 041K0JQ, 041K0JS, 041K0ZH, 041K0ZJ, 041K0ZK, 041K0ZL, 041K0ZM, 041K0ZN, 041K0ZP, 041K0ZQ, 041K0ZS, 041L09H, 041L09J, 041L09K, 041L09L, 041L09M, 041L09N, 041L09P, 041L09Q, 041L09S, 041L0JH, 041L0JJ, 041L0JK, 041L0JL, 041L0JM, 041L0JN, 041L0JP, 041L0JQ, 041L0JS, 041L0KH, 041L0KJ, 041L0KK, 041L0KL, 041L0KM, 041L0KN, 041L0KP, 041L0KQ, 041L0KS, 041L0ZH, 041L0ZJ, 041L0ZK, 041M0, 041M3, 041M4, 041N0, 041N3, 041N4, 041S0JQ, 041U0AP, 047C34, 047C35, 047C36, 047C37, 047D34, 047D35, 047D36, 047D37, 047H34, 047H35, 047H36, 047H37, 047J34, 047J35, 047J36, 047J37, 047K34, 047K35, 047K36, 047K37, 047L34, 047L35, 047L36, 047L37, 047M34, 047M35, 047M36, 047M37, 047N34, 047N35, 047N36, 047N37, 047P34, 047P35, 047P36, 047P37, 047Q34, 047Q35, 047Q36, 047Q37, 047R34, 047R35, 047R36, 047R37, 047S34, 047S35, 047S36, 047S37, 047T34, 047T35, 047T36, 047T37, 047U34, 047U35, 047U36, 047U37, 047V34, 047V35, 047V36, 047V37, 047W34, 047W35, 047W36, 047W37, 047Y34, 047Y35, 047Y36, 047Y37, 04C00ZZ, 04C03ZZ, 04CC0Z6, 04CC3Z6, 04CC4Z6, 04CD0, 04CD3, 04CD4, 04CH0, 04CH3, 04CH4, 04CJ0, 04CJ3, 04CJ4, 04CK0, 04CK3, 04CK4, 04CL0, 04CL3, 04CL4, 04CM0, 04CM3, 04CM4, 04CN0, 04CN3, 04CN4, 04CP0, 04CP3, 04CP4, 04CQ0, 04CQ3, 04CQ4, 04CR0, 04CR3, 04CR4, 04CS0, 04CS3, 04CS4, 04CT0, 04CT3, 04CT4, 04CU0, 04CU3, 04CU4, 04CV0, 04CV3, 04CV4, 04CY0, 04CY3, 04CY4, 0Y62, 0Y63, 0Y64, 0Y67, 0Y68 CPT: 0236T, 0237T, 0238T, 27590, 27591, 27592, 27598, 27888, 27889, 28800, 28805, 35302, 35303, 35304, 35305, 35306, 35331, 35351, 35355, 35361, 35363, 35371, 35372, 35381, 35452, 35454, 35456, 35459, 35470, 35472, 35473, 35474, 35483, 35492, 35493, 35495, 35521, 35533, 35537, 35538, 35539, 35540, 35548, 35556, 35558, 35563, 35565, 35566, 35571, 35583, 35585, 35587, 35621, 35623, 35637, 35638, 35646, 35647, 35654, 35656, 35661, 35663, 35665, 35666, 35671, 35700, 35876, 35879, 35881, 35883, 35884, 37184, 37185, 37186, 37205, 37206, 37207, 37208, 37220, 37221, 37222, 37223, 37224, 37225, 37226, 37227, 37228, 37229, 37230, 37231, 37232, 37233, 37234, 37235, 37236, 37237 | ICD-9-CM: 170.6, 170.7, 170.8, 170.9, 171.3, 172.7, 173.7, 198.5, 344.1, 711, 728.86, 733.2, 735, 735.1, 735.2, 735.3, 735.4, 735.5, 735.8, 735.9, 736.3, 736.31, 736.32, 736.39, 736.41, 736.42, 736.5, 736.6, 736.7, 736.71, 736.72, 736.73, 736.74, 736.75, 736.76, 736.79, 736.81, 736.89, 736.9, 754.3, 754.31, 754.32, 754.33, 754.35, 754.4, 754.41, 754.42, 754.43, 754.44, 754.5, 754.51, 754.52, 754.53, 754.59, 754.6, 754.61, 754.62, 754.69, 754.7, 754.71, 754.79, 755.02, 755.13, 755.14, 755.3, 755.4, 755.6, 755.61, 755.62, 755.63, 755.64, 755.65, 755.66, 755.67, 755.69, 755.8, 759.7, 759.89, 820, 820.1, 820.2, 820.3, 820.8, 820.9, 821, 821.1, 821.2, 821.3, 822, 822.1, 823, 823.1, 823.2, 823.3, 823.4, 823.8, 823.9, 824, 824.1, 824.2, 824.3, 824.4, 824.5, 824.6, 824.7, 824.8, 824.9, 825, 825.1, 825.2, 825.3, 826, 826.1, 827, 827.1, 828, 828.1, 829, 829.1, 835, 835.1, 836, 836.1, 836.2, 836.3, 836.4, 836.5, 836.6, 837, 837.1, 838, 838.1, 890, 890.1, 890.2, 891, 895, 895.1, 896, 896.1, 896.2, 896.3, 897, 897.1, 897.2, 897.3, 897.4, 897.5, 897.6, 897.7, 904, 904.1, 904.2, 904.3, 904.4, 904.5, 904.6, 904.7, 904.8, 904.9, 905.4, 928, 928.1, 928.2, 928.3, 928.8, 928.9, 929, 929.9, 959.6, 959.7, 996.4, 996.41, 996.42, 996.43, 996.44, 996.45, 996.46, 996.47, 996.49, 996.66, 996.67, 996.77, 996.78 ICD-10-CM: C40.20, C40.21, C40.22, C40.30, C40.31, C40.32, C41.4, C41.9, C43.70, C43.71, C43.72, C44.70, C44.71, C44.72, C44.79, C49.20, C49.21, C49.22, C79.51, C79.52, D03.70, D03.71, D03.72, E78.71, E78.72, G82.0, G82.1, G82.2, G82.3, G82.4, G82.5, G82.8, G82.9, M00.0, M00.1, M00.2, M00.8, M00.9, M20.0, M20.1, M20.10, M20.11, M20.12, M20.2, M20.20, M20.21, M20.22, M20.3, M20.30, M20.31, M20.32, M20.4, M20.5, M20.6, M20.60, M20.61, M20.62, M20.8, M20.9, M21.051, M21.052, M21.059, M21.061, M21.062, M21.069, M21.071, M21.072, M21.079, M21.151, M21.152, M21.159, M21.161, M21.162, M21.169, M21.171, M21.172, M21.179, M21.6X1, M21.6X2, M21.6X9, M21.70, M21.71, M21.72, M21.73, M21.74, M21.75, M21.76, M21.77, M21.79, M21.851, M21.852, M21.859, M21.861, M21.862, M21.869, M21.951, M21.952, M21.959, M21.961, M21.962, M21.969, M24.351, M24.352, M24.359, M24.361, M24.362, M24.369, M24.371, M24.372, M24.373, M24.374, M24.375, M24.376, M72, M84.0, M84.1, M84.2, M84.3, M84.4, M84.5, M84.6, M84.7, M84.8, M84.9, M85.651, M85.652, M85.659, M85.661, M85.662, M85.669, M85.671, M85.672, M85.679, M85.68, M85.69, Q65.0, Q65.00, Q65.01, Q65.02, Q65.1, Q65.2, Q65.3, Q65.30, Q65.31, Q65.32, Q65.4, Q65.5, Q65.6, Q65.8, Q65.81, Q65.82, Q65.89, Q65.9, Q66.30, Q66.31, Q66.32, Q66.40, Q66.41, Q66.42, Q66.50, Q66.51, Q66.52, Q66.70, Q66.71, Q66.72, Q66.80, Q66.81, Q66.82, Q66.89, Q69.2, Q70.20, Q70.21, Q70.22, Q70.23, Q70.30, Q70.31, Q70.32, Q70.33, Q72.00, Q72.01, Q72.02, Q72.03, Q72.10, Q72.11, Q72.12, Q72.13, Q72.20, Q72.21, Q72.22, Q72.23, Q72.30, Q72.31, Q72.32, Q72.33, Q72.40, Q72.41, Q72.42, Q72.43, Q72.50, Q72.51, Q72.52, Q72.53, Q72.60, Q72.61, Q72.62, Q72.63, Q72.70, Q72.71, Q72.72, Q72.73, Q72.80, Q72.81, Q72.82, Q72.83, Q72.89, Q72.90, Q72.91, Q72.92, Q72.93, Q74.8, Q74.9, Q87.2, Q87.3, Q87.5, Q87.81, Q87.82, Q87.89, Q89.7, Q89.8, S71.009A, S71.109A, S72.0, S72.00, S72.01, S72.02, S72.03, S72.04, S72.05, S72.06, S72.08, S72.09, S72.1, S72.10, S72.11, S72.12, S72.13, S72.14, S72.19, S72.2, S72.20, S72.21, S72.22, S72.23, S72.24, S72.25, S72.26, S72.3, S72.30, S72.31, S72.32, S72.33, S72.34, S72.35, S72.36, S72.39, S72.4, S72.40, S72.41, S72.42, S72.43, S72.44, S72.45, S72.46, S72.47, S72.49, S72.7, S72.70, S72.71, S72.8, S72.80, S72.81, S72.9, S72.90, S72.91, S72.92, S75.0, S75.00, S75.01, S75.02, S75.03, S75.04, S75.05, S75.06, S75.08, S75.09, S75.1, S75.10, S75.11, S75.12, S75.16, S75.18, S75.19, S75.2, S75.20, S75.21, S75.22, S75.26, S75.28, S75.29, S75.7, S75.8, S75.80, S75.81, S75.82, S75.83, S75.84, S75.85, S75.86, S75.88, S75.89, S75.9, S75.90, S75.91, S75.92, S75.93, S75.94, S75.95, S75.96, S75.98, S75.99, S77.0, S77.00, S77.01, S77.02, S77.1, S77.10, S77.11, S77.12, S77.2, S77.20, S77.21, S77.22, S79.0, S79.00, S79.01, S79.09, S79.1, S79.10, S79.11, S79.12, S79.13, S79.14, S79.19, S79.7, S79.8, S79.81, S79.82, S79.9, S79.91, S79.92, S81.009A, S81.809A, S82.0, S82.00, S82.01, S82.02, S82.03, S82.04, S82.09, S82.1, S82.10, S82.11, S82.12, S82.13, S82.14, S82.15, S82.16, S82.18, S82.19, S82.2, S82.20, S82.21, S82.22, S82.23, S82.24, S82.25, S82.26, S82.28, S82.29, S82.3, S82.30, S82.31, S82.32, S82.38, S82.39, S82.4, S82.40, S82.41, S82.42, S82.43, S82.44, S82.45, S82.46, S82.49, S82.5, S82.50, S82.51, S82.52, S82.53, S82.54, S82.55, S82.56, S82.6, S82.60, S82.61, S82.62, S82.63, S82.64, S82.65, S82.66, S82.7, S82.70, S82.71, S82.8, S82.80, S82.81, S82.82, S82.83, S82.84, S82.85, S82.86, S82.87, S82.88, S82.89, S82.9, S82.90, S82.91, S82.92, S83.0, S83.00, S83.01, S83.09, S83.1, S83.10, S83.11, S83.12, S83.13, S83.14, S83.18, S83.19, S83.2, S83.20, S83.21, S83.22, S83.23, S83.24, S83.25, S83.26, S83.27, S83.28, S83.29, S83.3, S83.30, S83.31, S83.32, S83.4, S83.40, S83.41, S83.42, S83.43, S83.49, S83.5, S83.50, S83.51, S83.52, S83.53, S83.59, S83.6, S83.60, S83.61, S83.62, S83.7, S83.8, S83.9, S83.90, S83.91, S83.92, S88.0, S88.1, S88.9, S89.0, S89.00, S89.01, S89.02, S89.03, S89.04, S89.09, S89.1, S89.10, S89.11, S89.12, S89.13, S89.14, S89.19, S89.2, S89.20, S89.21, S89.22, S89.29, S89.3, S89.30, S89.31, S89.32, S89.39, S89.7, S89.8, S89.80, S89.81, S89.82, S89.9, S89.90, S89.91, S89.92, S91.009A, S92.0, S92.00, S92.01, S92.02, S92.03, S92.04, S92.05, S92.06, S92.1, S92.10, S92.11, S92.12, S92.13, S92.14, S92.15, S92.19, S92.2, S92.20, S92.21, S92.22, S92.23, S92.24, S92.25, S92.28, S92.29, S92.3, S92.30, S92.31, S92.32, S92.33, S92.34, S92.35, S92.4, S92.40, S92.41, S92.42, S92.49, S92.5, S92.50, S92.51, S92.52, S92.53, S92.59, S92.7, S92.70, S92.71, S92.8, S92.81, S92.9, S92.90, S92.91, S93.0, S93.01, S93.02, S93.03, S93.04, S93.05, S93.06, S93.1, S93.10, S93.11, S93.12, S93.13, S93.14, S93.19, S93.2, S93.3, S93.30, S93.31, S93.32, S93.33, S93.38, S93.39, S93.4, S93.40, S93.41, S93.42, S93.43, S93.48, S93.49, S93.5, S93.50, S93.51, S93.52, S93.6, S93.60, S93.61, S93.62, S93.69, S98.0, S98.1, S98.2, S98.3, S98.4, S98.9, S99.0, S99.00, S99.01, S99.02, S99.03, S99.04, S99.09, S99.1, S99.10, S99.11, S99.12, S99.13, S99.14, S99.19, S99.2, S99.20, S99.21, S99.22, S99.23, S99.24, S99.29, S99.7, S99.8, S99.81, S99.82, S99.9, S99.91, S99.92, T84.0, T84.01, T84.02, T84.03, T84.04, T84.05, T84.06, T84.09, T84.1, T84.11, T84.12, T84.19, T84.2, T84.21, T84.22, T84.29, T84.3, T84.31, T84.32, T84.39, T84.4, T84.41, T84.42, T84.49, T84.5, T84.50, T84.51, T84.52, T84.53, T84.54, T84.59, T84.6, T84.60, T84.61, T84.62, T84.63, T84.69, T84.7, T84.8, T84.81, T84.82, T84.83, T84.84, T84.85, T84.86, T84.89, T84.9, Z89.61, Z89.62 |
|  |  |
|  |  |
|  |  |
|  |  |
|  |  |
|  |  |
|  |  |
|  |  |
|  |  |
|  |  |
|  |  |
|  |  |
|  |  |
|  |  |
|  |  |
|  |  |
|  |  |
|  |  |
|  |  |
|  |  |
|  |  |
|  |  |
|  |  |
|  |  |
|  |  |
|  |  |
|  |  |
|  |  |
|  |  |
|  |  |
|  |  |
|  |  |
|  |  |
|  |  |
|  |  |
|  |  |
|  |  |
|  |  |
|  |  |
|  |  |
|  |  |
|  |  |
|  |  |
|  |  |
|  |  |
|  |  |
|  |  |
|  |  |
|  |  |
|  |  |
|  |  |
|  |  |
|  |  |
|  |  |
|  |  |
|  |  |
|  |  |
|  |  |
|  |  |
|  |  |
|  |  |
|  |  |
|  |  |
|  |  |
|  |  |
|  |  |
|  |  |
|  |  |
|  |  |
|  |  |
|  |  |
|  |  |
|  |  |
|  |  |
|  |  |
|  |  |
|  |  |
|  |  |
|  |  |
|  |  |
|  |  |
|  |  |
|  |  |
|  |  |
|  |  |
|  |  |
|  |  |
|  |  |
|  |  |
|  |  |
|  |  |
|  |  |
|  |  |
|  |  |
|  |  |
|  |  |
|  |  |

**eTable 2. Baseline Demographic and Clinical Characteristics Stratified by Race/Ethnicity and Peripheral Artery Disease (PAD) Status (PAD, Non-PAD, and Overall): American Family Cohort, Jan 1, 2018, to Dec 31, 2022 (n=2,313,650)**

| **Characteristics by Race/Ethnicity** | **All** | **PAD** | **Non-PAD** |
| --- | --- | --- | --- |
| Total across all race/ethnicity groups, n | 2,313,650 | 18,405 | 2,295,245 |
| **Non-Hispanic Black or African American** | | | |
| Total, n | 158,746 | 1,918 | 156,828 |
| Age, yr (mean, SD) | 62 (12.6) | 70.2 (10.5) | 61.9 (12.6) |
| Female, n (%) | 98,612 (62) | 1,123 (59) | 97,489 (62) |
| Chronic conditions, n (%) |  |  |  |
| Cerebrovascular Disease (CVD) | 5,826 (4) | 161 (8) | 5,665 (4) |
| Coronary Artery Disease (CAD) | 9,762 (6) | 319 (17) | 9,443 (6) |
| Heart Failure (HF) | 8,917 (6) | 209 (11) | 8,708 (6) |
| Hypertension (HTN) | 107,032 (67) | 1,663 (87) | 105,369 (67) |
| Diabetes | 46,315 (29) | 834 (43) | 45,481 (29) |
| Hyperlipidemia (HLD) | 77,633 (49) | 1,236 (64) | 76,397 (49) |
| Body Mass Index (BMI), mean (SD) | 32.0 (6.9) | 30.2 (6.4) | 32.0 (6.9) |
| Current smokers, n (%) | 18,764 (12) | 342 (18) | 18,422 (12) |
| **Non-Hispanic White** | | | |
| Total, n | 1,584,560 | 12,374 | 1,572,186 |
| Age, yr (mean, SD) | 65.1 (13.2) | 72.8 (10.3) | 65 (13.2) |
| Female, n (%) | 890,759 (56) | 6,082 (49) | 884,677 (56) |
| Chronic conditions, n (%) |  |  |  |
| CVD | 58,777 (4) | 1,302 (11) | 57,475 (4) |
| CAD | 140,494 (9) | 3,051 (25) | 137,443 (9) |
| HF | 69,063 (4) | 1,350 (11) | 67,713 (4) |
| HTN | 842,935 (53) | 8,975 (73) | 833,960 (53) |
| Diabetes | 289,169 (18) | 3,775 (31) | 285,394 (18) |
| HLD | 830,565 (52) | 8,278 (67) | 822,287 (52) |
| BMI, mean (SD) | 30.3 (6.6) | 29.9 (6.3) | 30.3 (6.6) |
| Current smokers, n (%) | 179,286 (11) | 2,365 (19) | 175,921 (11) |
| **Hispanic** | | | |
| Total, n | 170,758 | 1,746 | 169,012 |
| Age, yr (mean, SD) | 60.5 (12.9) | 71.4 (10.3) | 60.4 (12.9) |
| Female, n (%) | 98,764 (58) | 946 (54) | 97,818 (58) |
| Chronic conditions, n (%) |  |  |  |
| CVD | 4,070 (2) | 112 (6) | 3,958 (2) |
| CAD | 9,869 (6) | 296 (17) | 9,573 (6) |
| HF | 5,816 (3) | 177 (10) | 5,639 (3) |
| HTN | 87,233 (51) | 1,334 (76) | 85,899 (51) |
| Diabetes | 49,892 (29) | 796 (46) | 49,096 (29) |
| HLD | 91,806 (54) | 1,173 (67) | 90,633 (54) |
| BMI, mean (SD) | 30.6 (6.1) | 32.0 (6.6) | 30.6 (6.1) |
| Current smokers, n (%) | 12,154 (7) | 146 (8) | 12,008 (7) |
| **Asian** | | | |
| Total, n | 41,886 | 253 | 41,633 |
| Age, yr (mean, SD) | 61.9 (13.3) | 71.3 (10.9) | 61.9 (13.3) |
| Female, n (%) | 24,619 (59) | 133 (53) | 24,486 (59) |
| Chronic conditions, n (%) |  |  |  |
| CVD | 998 (2) | 23 (9) | 975 (2) |
| CAD | 2,339 (6) | 46 (18) | 2,293 (6) |
| HF | 916 (2) | 18 (7) | 898 (2) |
| HTN | 20,508 (49) | 188 (74) | 20,320 (49) |
| Diabetes | 9,973 (24) | 95 (38) | 9,878 (24) |
| HLD | 23,063 (55) | 185 (73) | 22,878 (55) |
| BMI, mean (SD) | 26.6 (5.1) | 25.9 (4.9) | 26.6 (5.1) |
| Current smokers, n (%) | 2,089 (5) | 25 (10) | 2,064 (5) |
| **American Indian or Alaska Native** | | | |
| Total, n | 10,578 | 74 | 10,504 |
| Age, yr (mean, SD) | 60.7 (12.4) | 68.9 (10.2) | 60.7 (12.4) |
| Female, n (%) | 5,843 (55) | 35 (47) | 5,808 (55) |
| Chronic conditions, n (%) |  |  |  |
| CVD | 369 (3) | 10 (14) | 359 (3) |
| CAD | 838 (8) | 18 (24) | 820 (8) |
| HF | 500 (5) | 9 (12) | 491 (5) |
| HTN | 5,024 (47) | 47 (64) | 4,977 (47) |
| Diabetes | 1,813 (17) | 24 (32) | 1,789 (17) |
| HLD | 3,247 (31) | 41 (55) | 3,206 (31) |
| BMI, mean (SD) | 31.5 (7.1) | NA | 31.5 (7.1) |
| Current smokers, n (%) | 1,627 (15) | 13 (18) | 1,614 (15) |
| **Native Hawaiian or Other Pacific Islander** | | | |
| Total, n | 4,367 | 37 | 4,330 |
| Age, yr (mean, SD) | 61 (12.9) | 69.5 (9) | 60.9 (12.9) |
| Female, n (%) | 2,519 (58) | 14 (38) | 2,505 (58) |
| Chronic conditions, n (%) |  |  |  |
| CVD | 128 (3) | 4 (11) | 124 (3) |
| CAD | 320 (7) | 5 (14) | 315 (7) |
| HF | 237 (5) | 9 (24) | 228 (5) |
| HTN | 2,251 (52) | 25 (68) | 2,226 (51) |
| Diabetes | 1,024 (23) | 19 (51) | 1,005 (23) |
| HLD | 2,116 (48) | 24 (65) | 2,092 (48) |
| BMI, mean (SD) | 32.6 (7.4) | 32.6 (7.5) | 32.6 (7.4) |
| Current smokers, n (%) | 450 (10) | 6 (16) | 444 (10) |
| **Other/Unknown** | | | |
| Total, n | 342,755 | 2,003 | 340,752 |
| Age, yr (mean, SD) | 63.1 (13.3) | 71.1 (10.8) | 63.1 (13.3) |
| Female, n (%) | 194,184 (57) | 992 (50) | 193,192 (57) |
| Chronic conditions, n (%) |  |  |  |
| CVD | 8,769 (3) | 173 (9) | 8,596 (3) |
| CAD | 19,514 (6) | 335 (17) | 19,179 (6) |
| HF | 10,604 (3) | 185 (9) | 10,419 (3) |
| HTN | 142,846 (42) | 1,325 (66) | 141,521 (42) |
| Diabetes | 55,366 (16) | 607 (30) | 54,759 (16) |
| HLD | 134,633 (39) | 1,114 (56) | 133,519 (39) |
| BMI, mean (SD) | 29.8 (6.6) | 28.2 (5.7) | 29.8 (6.6) |
| Current smokers, n (%) | 33,288 (10) | 306 (15) | 32,982 (10) |

**eTable 3. Interaction Effects of Race/Ethnicity and Gender and Chronic Conditions on Peripheral Artery Disease (PAD) from an Age-Adjusted Logit Probability Model** **: American Family Cohort, Jan 1, 2018, to Dec 31, 2022 (n=2,313,650)**

| **Variable** | **Coefficient**  **(95% CI)** | **P value** |
| --- | --- | --- |
| **Cerebrovascular Disease (CVD)** | | |
| Non-Hispanic White: Non-CVD | Reference | |
| Non-Hispanic Black or African American: Has CVD | -0.17 (-0.35,0) | 0.06 |
| Hispanic: Has CVD | -0.13 (-0.34,0.07) | 0.2 |
| **Coronary Artery Disease (CAD)** | | |
| Non-Hispanic White: Non-CAD | Reference | |
| Non-Hispanic Black or African American: Has CAD | -0.09 (-0.22,0.04) | 0.2 |
| Hispanic: Has CAD | -0.12 (-0.25,0.02) | 0.09 |
| **Heart Failure (HF)** | | |
| Non-Hispanic White: Non-HF | Reference | |
| Non-Hispanic Black or African American: Has HF | -0.15 (-0.30,0.01) | 0.07 |
| Hispanic: Has HF | 0.09 (-0.08,0.25) | 0.3 |
| **Hypertension (HTN)** | | |
| Non-Hispanic White: Non-HTN | Reference | |
| Non-Hispanic Black or African American: Has HTN | 0.33 (0.19,0.47) | <0.001 |
| Hispanic: Has HTN | 0.20 (0.09,0.32) | <0.001 |
| **Diabetes** | | |
| Non-Hispanic White: Non-Diabetes | Reference | |
| Non-Hispanic Black or African American: Has Diabetes | -0.09 (-0.19,0.01) | 0.08 |
| Hispanic: Has Diabetes | -0.06 (-0.16,0.05) | 0.3 |
| **Hyperlipidemia (HLD)** | | |
| Non-Hispanic White: Non-HLD | Reference | |
| Non-Hispanic Black or African American: Has HLD | 0.02 (-0.08,0.12) | 0.7 |
| Hispanic: Has HLD | -0.08 (-0.18,0.03) | 0.2 |
| Gender |  |  |
| Non-Hispanic White: Male | Reference | |
| Non-Hispanic Black or African American: Female | 0.13 (0.03,0.23) | 0.008 |
| Hispanic: Female | 0.16 (0.06,0.26) | 0.002 |

**eTable 4. Age-Adjusted Risk Ratios and 95% Confidence Intervals (CI) for Peripheral Artery Disease (PAD) by Chronic Conditions Across Race and Ethnicity Categories: American Family Cohort, Jan 1, 2018, to Dec 31, 2022 (n=2,313,650). *P<0.05, **P<0.01, ***P<0.001.**

| **Chronic Conditions^#^** | **Non-Hispanic Black or African American** | | **Non-Hispanic White** | | **Hispanic** | |
| --- | --- | --- | --- | --- | --- | --- |
|  | **Risk Ratio (95% CI)^##^** | **P Value** | **Risk Ratio (95% CI)** | **P Value** | **Risk Ratio (95% CI)** | **P Value** |
| CVD vs. Non-CVD | 1.67 (1.42,1.96) | <0.001*** | 1.98 (1.87,2.10) | <0.001*** | 1.74 (1.44,2.10) | <0.001*** |
| CAD vs. Non-CAD | 2.12 (1.88,2.39) | <0.001*** | 2.33 (2.24,2.43) | <0.001*** | 2.06 (1.82,2.34) | <0.001*** |
| HF vs  Non-HF | 1.40 (1.21,1.61) | <0.001*** | 1.62 (1.53,1.72) | <0.001*** | 1.75 (1.50,2.04) | <0.001*** |
| HTN vs. Non-HTN | 2.37 (2.08,2.70) | <0.001*** | 1.72 (1.65,1.79) | <0.001*** | 2.10 (1.88,2.34) | <0.001*** |
| Diabetes vs. Non-Diabetes | 1.61 (1.47,1.76) | <0.001*** | 1.76 (1.70,1.83) | <0.001*** | 1.67 (1.52,1.83) | <0.001*** |
| HLD vs. Non-HLD | 1.48 (1.35,1.63) | <0.001*** | 1.46 (1.41,1.52) | <0.001*** | 1.35 (1.23,1.50) | <0.001*** |

^#^Chronic conditions include Cerebrovascular Disease (CVD), Coronary Artery Disease (CAD), Heart Failure (HF), Hypertension (HTN), Diabetes, and Hyperlipidemia (HLD).
^##^Risk ratios were calculated from a multivariate log-binomial regression with predictors of age, race and ethnicity category, a chronic condition, and an interaction between the race and ethnicity category and the chronic condition.

**eTable 5. Age-Adjusted Risk Ratios and 95% Confidence Intervals (CIs) for Peripheral Artery Disease (PAD) by Each of the Chronic Conditions Across Race and Ethnicity Categories: American Family Cohort, Jan 1, 2018, to Dec 31, 2022 (n=2,313,650). *P<0.05, **P<0.01, ***P<0.001.**

| **Chronic Conditions^#^** | **Non-Hispanic Black or African American vs. Non-Hispanic White** | | **Hispanic vs. Non-Hispanic White** | | **Hispanic vs. Non-Hispanic Black or African American** | |
| --- | --- | --- | --- | --- | --- | --- |
|  | **Risk Ratio (95% CI)^##^** | **P Value** | **Risk Ratio (95% CI)** | **P Value** | **Risk Ratio (95% CI)** | **P Value** |
| Non-CVD | 1.82 (1.71,1.93) | <0.001*** | 1.64 (1.54,1.75) | <0.001*** | 0.90 (0.83,0.98) | 0.03* |
| CVD | 1.53 (1.26,1.85) | <0.001*** | 1.44 (1.14,1.80) | 0.001** | 0.94 (0.71,1.25) | 0.9 |
| Non-CAD | 1.87 (1.76,2.00) | <0.001*** | 1.68 (1.57,1.79) | <0.001*** | 0.89 (0.82,0.97) | 0.02* |
| CAD | 1.70 (1.49,1.95) | <0.001*** | 1.48 (1.29,1.71) | <0.001*** | 0.87 (0.72,1.05) | 0.5 |
| Non-HF | 1.80 (1.70,1.91) | <0.001*** | 1.59 (1.50,1.70) | <0.001*** | 0.89 (0.82,0.96) | 0.006** |
| HF | 1.55 (1.31,1.84) | <0.001*** | 1.72 (1.43,2.06) | <0.001*** | 1.11 (0.87,1.40) | 0.9 |
| Non-HTN | 1.26 (1.09,1.47) | 0.004** | 1.36 (1.20,1.54) | <0.001*** | 1.08 (0.89,1.30) | 0.9 |
| HTN | 1.74 (1.64,1.85) | <0.001*** | 1.66 (1.55,1.78) | <0.001*** | 0.95 (0.87,1.04) | 0.8 |
| Non-Diabetes | 1.72 (1.60,1.85) | <0.001*** | 1.51 (1.40,1.64) | <0.001*** | 0.88 (0.79,0.98) | 0.04* |
| Diabetes | 1.57 (1.44,1.72) | <0.001*** | 1.43 (1.30,1.56) | <0.001*** | 0.91 (0.81,1.02) | 0.4 |
| Non-HLD | 1.78 (1.62,1.97) | <0.001*** | 1.67 (1.51,1.86) | <0.001*** | 0.94 (0.82,1.07) | 0.9 |
| HLD | 1.81 (1.69,1.95) | <0.001*** | 1.55 (1.44,1.67) | <0.001*** | 0.85 (0.78,0.94) | 0.001** |

^#^Chronic conditions include Cerebrovascular Disease (CVD), Coronary Artery Disease (CAD), Heart Failure (HF), Hypertension (HTN), Diabetes, and Hyperlipidemia (HLD).

^##^Risk ratios were calculated from a multivariate log-binomial regression with predictors of age, race and ethnicity category, a chronic condition, and an interaction between the race and ethnicity category and the chronic condition.

**eTable 6** **. Baseline Social Deprivation Characteristics Stratified by Race/Ethnicity and Peripheral Artery Disease (PAD) Status (PAD, Non-PAD, and Overall): American Family Cohort, Jan 1, 2018, to Dec 31, 2022 (n=2,313,650)**

| **Characteristics by race/ethnicity** | **All** | **PAD** | **Non-PAD** |
| --- | --- | --- | --- |
| Total across all race/ethnicity groups, n | 2,313,650 | 18,405 | 2,295,245 |
| **Non-Hispanic Black or African American** | | | |
| Social Deprivation, median (IQR) | | | |
| Social Deprivation Index (SDI) at census tract level | 60.4 (27.3) | 67.3 (25.6) | 60.3 (27.3) |
| Rural-Urban-Commuting Area (RUCA) at zip code level, n (%) | | | |
| Metropolitan areas | 128,683 (81) | 1,562 (81) | 127,121 (81) |
| Micropolitan areas | 17,196 (11) | 212 (11) | 16,984 (11) |
| Small town | 9,548 (6) | 99 (5) | 9,449 (6) |
| Rural areas | 2,786 (2) | 28 (1) | 2,758 (2) |
| Unknown | 533 (0.3) | 17 (1) | 516 (0.3) |
| **Non-Hispanic White** | | | |
| Social Deprivation, median (IQR) | | | |
| Social Deprivation Index (SDI) at census tract level | 40.1 (25) | 45.7 (25) | 40.1 (25) |
| Rural-Urban-Commuting Area (RUCA) at zip code level, n (%) | | | |
| Metropolitan areas | 1,081,230 (68) | 8,265 (67) | 1,072,965 (68) |
| Micropolitan areas | 261,599 (17) | 2,040 (16) | 259,559 (17) |
| Small town | 143,129 (9) | 1,084 (9) | 142,045 (9) |
| Rural areas | 93,256 (6) | 956 (8) | 92,300 (6) |
| Unknown | 5,346 (0.3) | 29 (0.2) | 5,317 (0.3) |
| **Hispanic** | | | |
| Social Deprivation, median (IQR) | | | |
| Social Deprivation Index (SDI) at census tract level | 59.5 (27.4) | 64.5 (27.6) | 59.4 (27.4) |
| Rural-Urban-Commuting Area (RUCA) at zip code level, n (%) | | | |
| Metropolitan areas | 132,612 (78) | 1,513 (87) | 131,099 (78) |
| Micropolitan areas | 21,336 (12) | 113 (6) | 21,223 (13) |
| Small town | 11,528 (7) | 79 (5) | 11,449 (7) |
| Rural areas | 4,247 (2) | 29 (2) | 4,218 (2) |
| Unknown | 1,035 (1) | 12 (1) | 1,023 (1) |
| **Asian** | | | |
| Social Deprivation, median (IQR) | | | |
| Social Deprivation Index (SDI) at census tract level | 41.7 (28) | 47.4 (28.5) | 41.7 (28) |
| Rural-Urban-Commuting Area (RUCA) at zip code level, n (%) | | | |
| Metropolitan areas | 38,515 (92) | 248 (98) | 38,267 (92) |
| Micropolitan areas | 2,480 (6) | 4 (2) | 2,476 (6) |
| Small town | 556 (1) | 1 (0.4) | 555 (1) |
| Rural areas | 189 (0.5) | 0 | 189 (0.5) |
| Unknown | 146 (0.3) | 0 | 146 (0.4) |
| **American Indian or Alaska Native** | | | |
| Social Deprivation, median (IQR) | | | |
| Social Deprivation Index (SDI) at census tract level | 42.8 (28) | 49.3 (31) | 42.7 (28) |
| Rural-Urban-Commuting Area (RUCA) at zip code level, n (%) | | | |
| Metropolitan areas | 4,430 (42) | 44 (59) | 4,386 (42) |
| Micropolitan areas | 1,245 (12) | 12 (16) | 1,233 (12) |
| Small town | 978 (9) | 8 (11) | 970 (9) |
| Rural areas | 1,150 (11) | 10 (14) | 1,140 (11) |
| Unknown | 2,775 (26) | 0 | 2,775 (26) |
| **Native Hawaiian or Other Pacific Islander** | | | |
| Social Deprivation, median (IQR) | | | |
| Social Deprivation Index (SDI) at census tract level | 43.2 (27.4) | 41.8 (24.7) | 43.2 (27.4) |
| Rural-Urban-Commuting Area (RUCA) at zip code level, n (%) | | | |
| Metropolitan areas | 3,177 (73) | 30 (81) | 3,147 (73) |
| Micropolitan areas | 913 (21) | 3 (8) | 910 (21) |
| Small town | 180 (4) | 0 | 180 (4) |
| Rural areas | 78 (2) | 4 (11) | 74 (2) |
| Unknown | 19 (0.4) | 0 | 19 (0.4) |
| **Other/Unknown** | | | |
| Social Deprivation, median (IQR) | | | |
| Social Deprivation Index (SDI) at census tract level | 42.7 (27.3) | 51.6 (28.5) | 42.6 (27.3) |
| Rural-Urban-Commuting Area (RUCA) at zip code level, n (%) | | | |
| Metropolitan areas | 263,903 (77) | 1,623 (81) | 262,280 (77) |
| Micropolitan areas | 37,927 (11) | 190 (9) | 37,737 (11) |
| Small town | 21,508 (6) | 85 (4) | 21,423 (6) |
| Rural areas | 13,075 (4) | 96 (5) | 12,979 (4) |
| Unknown | 6,342 (2) | 9 (0.4) | 6,333 (2) |

**eTable 7** **a. Age-Adjusted Odds Ratios and 95% Confidence Intervals (CI) for Peripheral Artery Disease (PAD) Across Social Deprivation Variables Among Non-Hispanic Black or African American, Non-Hispanic White, and Hispanic Patients: American Family Cohort, Jan 1, 2018, to Dec 31, 2022 (n=2,313,650). *P<0.05, **P<0.01, ***P<0.001**

| **Characteristics** | **Non-Hispanic Black or African American** | | **Non-Hispanic White** | | **Hispanic** | |
| --- | --- | --- | --- | --- | --- | --- |
|  | **Odds Ratio (95% CI)** | **P Value** | **Odds Ratio (95% CI)** | **P Value** | **Odds Ratio (95% CI)** | **P Value** |
| Social Deprivation Index (SDI) Quintile^#^ at census tract level | | | | | | |
| Quintile 1: 0-18 | Reference | <0.001  *** | Reference | <0.001  *** | Reference | <0.001  *** |
| Quintile 2: 19-37 | 1.24 (0.96,1.60) |  | 1.14 (1.07,1.22) |  | 0.95 (0.76,1.20) |  |
| Quintile 3: 38-57 | 1.41 (1.11,1.79) |  | 1.43 (1.35,1.52) |  | 0.94 (0.76,1.17) |  |
| Quintile 4: 58-77 | 1.61 (1.28,2.02) |  | 1.55 (1.45,1.65) |  | 1.02 (0.83,1.24) |  |
| Quintile 5: 78-100 | 2.06 (1.66,2.56) |  | 1.87 (1.74,2.02) |  | 1.30 (1.08,1.56) |  |
| Rural-Urban-Commuting Area (RUCA) at zip code level | | | | | | |
| Metropolitan areas | Reference | 0.05 | Reference | <0.001  *** | Reference | <0.001  *** |
| Micropolitan areas | 0.98 (0.85,1.13) |  | 0.99 (0.94,1.04) |  | 0.48 (0.40,0.58) |  |
| Small town | 0.81 (0.66,0.99) |  | 0.96 (0.91,1.03) |  | 0.53 (0.43,0.67) |  |
| Rural areas | 0.71 (0.49,1.03) |  | 1.24 (1.15,1.32) |  | 0.51 (0.36,0.74) |  |

^#^SDI quintiles were calculated based on SDI distribution of the population from 2016-2020 American Community Survey 5-Year Estimates.

**eTable 7** **b. Age-Adjusted Odds Ratios and 95% Confidence Intervals (CI) for Peripheral Artery Disease (PAD) Across Social Deprivation Variables Among Asian, American Indican or Alaska Native, and Native Hawaiian or Other Pacific Islander patients: American Family Cohort, Jan 1, 2018, to Dec 31, 2022 (n=2,313,650). *P<0.05, **P<0.01, ***P<0.001**

| **Characteristics** | **Asian** | | **American Indian or Alaska Native** | | **Native Hawaiian or Other Pacific Islander** | |
| --- | --- | --- | --- | --- | --- | --- |
|  | **Odds Ratio (95% CI)** | **P Value** | **Odds Ratio (95% CI)** | **P Value** | **Odds Ratio (95% CI)** | **P Value** |
| Social Deprivation Index (SDI) Quintile^#^ at census tract level | | | | | | |
| Quintile 1: 0-18 | Reference | 0.5 | Reference | 0.3 | Reference | 0.7 |
| Quintile 2: 19-37 | 1.19 (0.79,1.79) |  | 0.73 (0.29,1.83) |  | 1.87 (0.66,5.29) |  |
| Quintile 3: 38-57 | 1.16 (0.77,1.74) |  | 0.82 (0.34,2.00) |  | 1.72 (0.61,4.86) |  |
| Quintile 4: 58-77 | 1.29 (0.87,1.90) |  | 1.08 (0.50,2.34) |  | 1.06 (0.32,3.48) |  |
| Quintile 5: 78-100 | 1.46 (0.97,2.19) |  | 1.88 (0.87,4.10) |  | 1.26 (0.35,4.51) |  |
| Rural-Urban-Commuting Area (RUCA) at zip code level | | | | | | |
| Metropolitan areas | Reference | <0.001 *** | Reference | 0.9 | Reference | 0.001 * |
| Micropolitan areas | 0.21 (0.08,0.57) |  | 0.93 (0.49,1.77) |  | 0.33 (0.10,1.09) |  |
| Small town | 0.30 (0.04,2.18) |  | 0.87 (0.41,1.85) |  | 0  (0,Inf) |  |
| Rural areas | 0  (0,Inf) |  | 0.92 (0.46,1.83) |  | 5.43 (1.85,15.95) |  |

^#^SDI quintiles were calculated based on SDI distribution of the population from 2016-2020 American Community Survey 5-Year Estimates.

**eFigure 1. A flow chart of the PAD (top) and the non-PAD (bottom) cohort.**

**
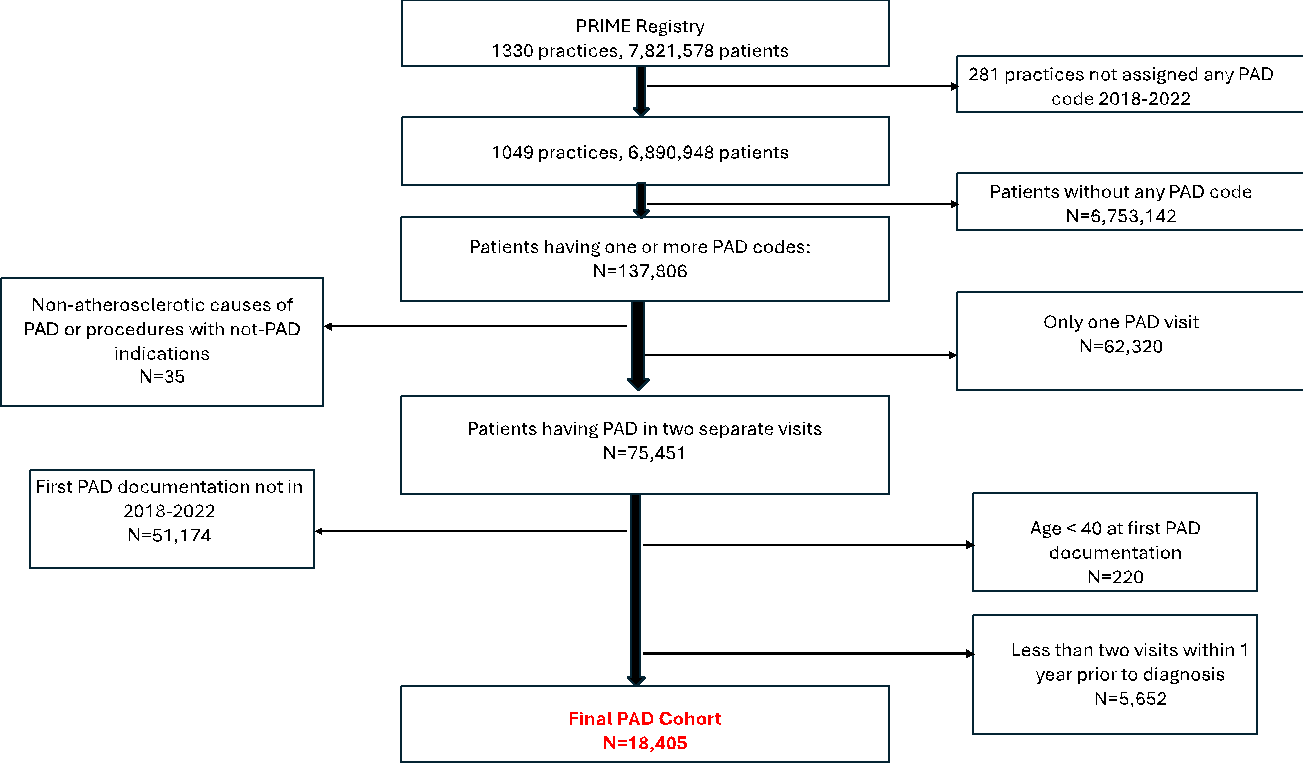
**

**
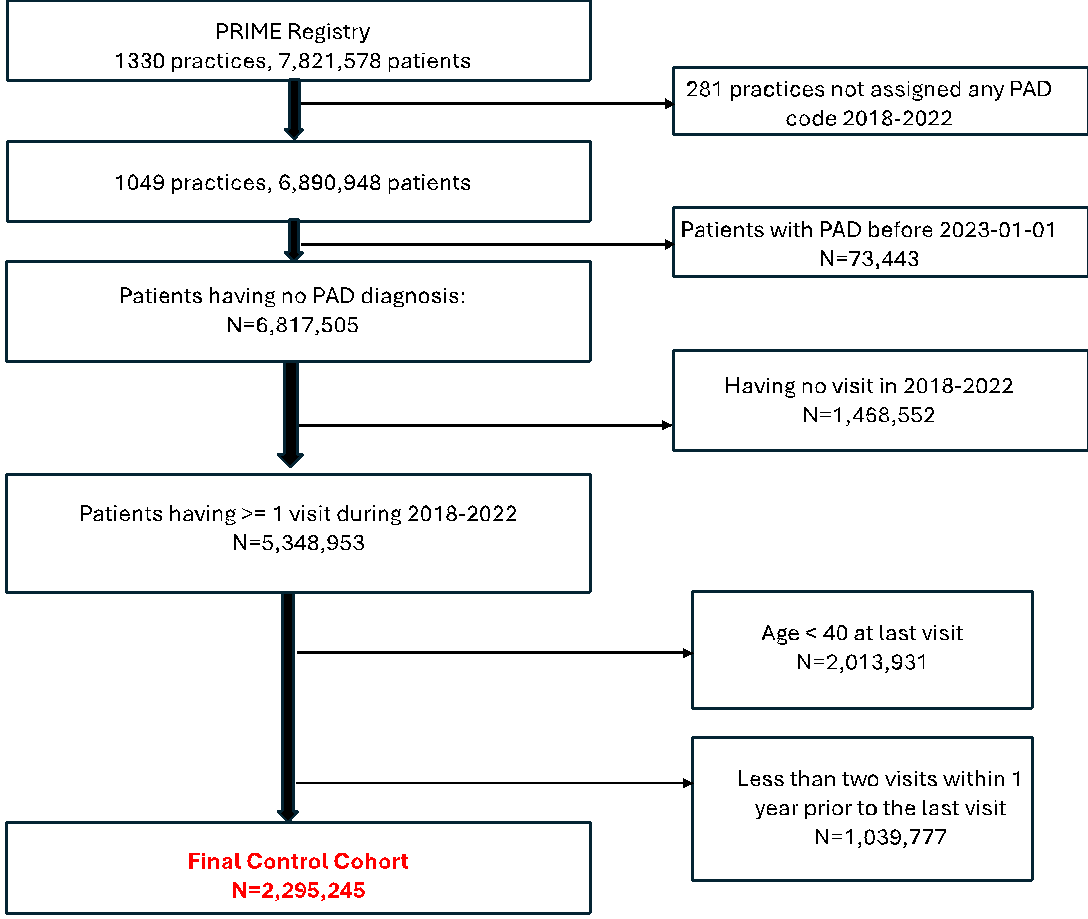
**

**eFigure 2. Social Deprivation Index (SDI) Distribution by Race/Ethnicity (Non-Hispanic Black or African American, Non-Hispanic White, Hispanic) for All Patients (Left) and Patients with Peripheral Artery Disease (Right): American Family Cohort, Jan 1, 2018, to Dec 31, 2022**


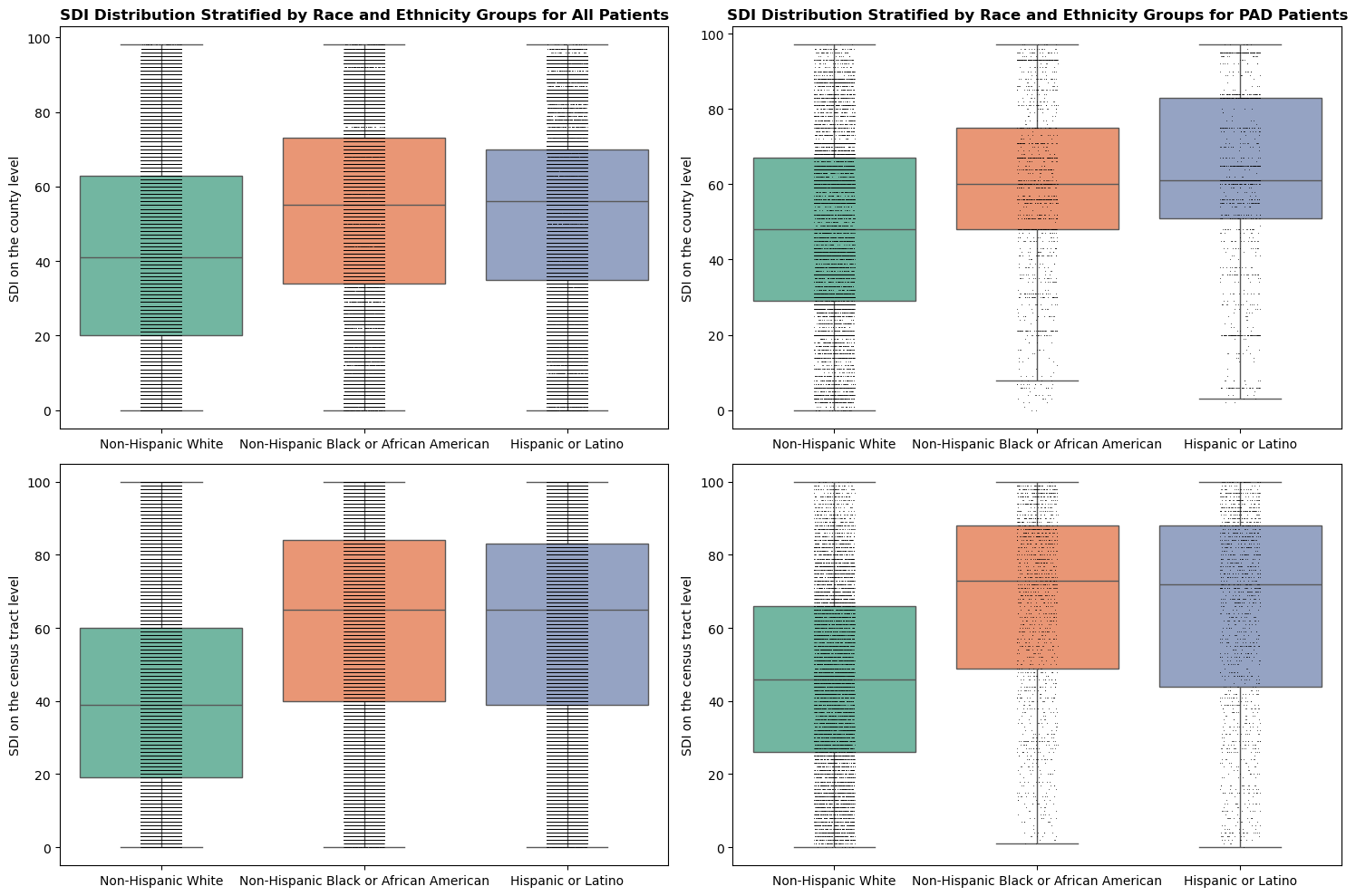


**eFigure 3. Age-Adjusted Annual Cumulative Incidence Rates of New Cases of Peripheral Arterial Disease (PAD), Coronary Artery Disease (CAD), and Hyperlipidemia (HLD) per 1,000 Individuals: American Family Cohort, Jan 1, 2018, to Dec 31, 2022**


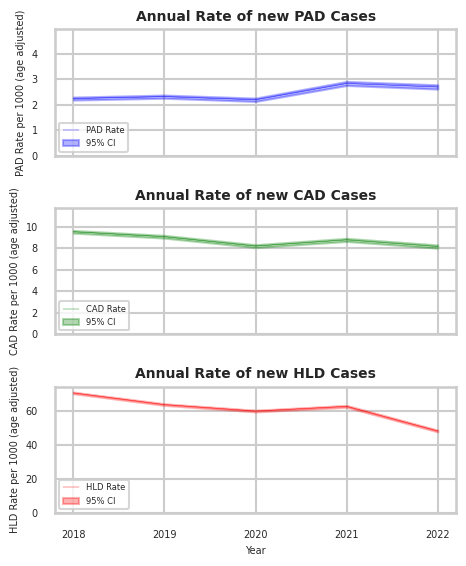

Supplement: Supplementary material [file NIHMS2196453-supplement-Supplementary_material.docx]
